# Supplementary material for: PI3K/AKT signaling allows for MAPK/ERK pathway independency mediating dedifferentiation-driven treatment resistance in melanoma
Source: Cell Commun Signal. 2022 Nov 24;20:187. doi: 10.1186/s12964-022-00989-y (PMC9700886; doi:10.1186/s12964-022-00989-y)
Supplement: Supplementary file 11 — Additional file 10. Fig. S1. Cell cycle analysis and sensitivity to inhibitors in BRAFV600E mutant MaMel cells. Fig. S2. Apoptotic and proliferative response of BRAFV600E mutant MaMel cells under inhibition. Fig. S3. MITF/AXL ratios in BRAFV600E mutant MaMel cells. Fig. S4. Over-represented targets of transcription factors within groups of genes correlated with the degree of resistance in BRAFV600E mutant MaMel cells. Fig. S5. Transcriptome profiles of melanoma cells with different NRAS/BRAF mutation status and degrees of differentiation. Fig. S6. Apoptotic and proliferative response of MaMel cells under combined BRAF and ABC transporter inhibition. Fig. S7. PTEN and FOXO1 expression in BRAFV600E mutant MaMel cells. [file 12964_2022_989_MOESM11_ESM.pdf]

Supplementary figures for

PI3K/AKT signaling allows for MAPK/ERK pathway independency mediating dedifferentiation-driven treatment resistance in melanoma

Eyleen Corrales[1,2,3†], Ella Levit-Zerdoun[1,2,4,5†], Patrick Metzger[1,2†], Ralf Mertes[1,2], Ariane Lehmann[1,2], Julia Münch[1], Steffen Lemke[1], Silke Kowar[1,2], and Melanie Boerries[1,2,4,5*]

[1] Institute of Molecular Medicine and Cell Research (IMMZ), University of Freiburg, Stefan-Meier-Str. 17, 79104 Freiburg, Germany.

[2] Institute of Medical Bioinformatics and Systems Medicine (IBSM), Medical Center-University of Freiburg, Faculty of Medicine, University of Freiburg, Breisacherstr. 153, 79110 Freiburg, Germany.

[3] Faculty of Biology, University of Freiburg, Schänzlestr. 1, 79104 Freiburg,Germany.

[4] German Cancer Research Center (DKFZ), Im Neuenheimer Feld 280, 69120 Heidelberg, Germany.

[5] German Cancer Consortium (DKTK), Freiburg, Germany.

† These authors share first authorship

* Corresponding author

Fig. S1

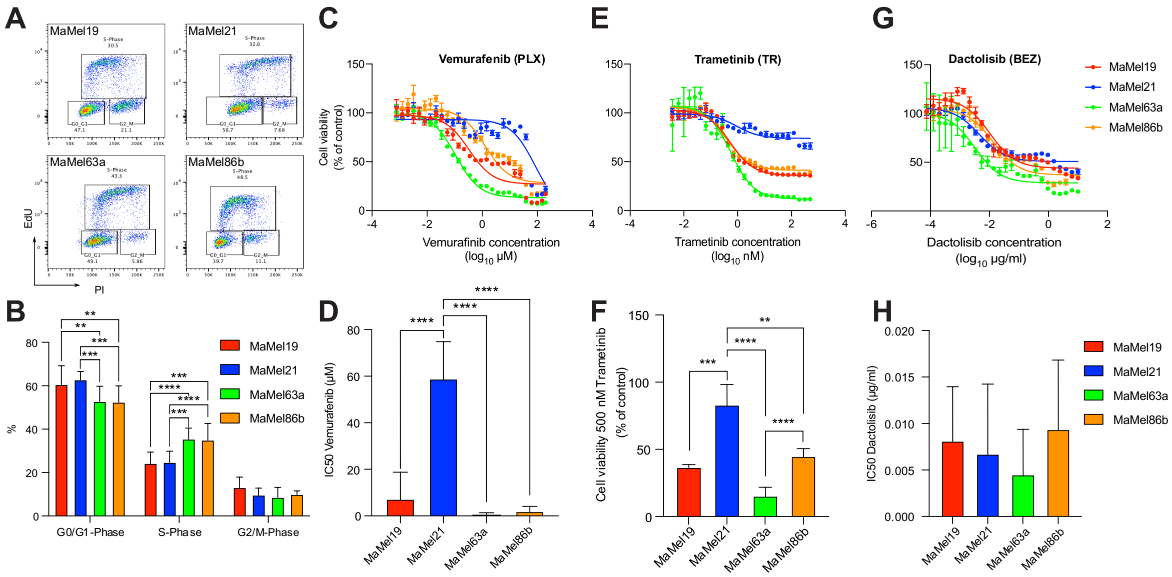

Fig. S1. Cell cycle analysis and sensitivity to inhibitors in BRAF^{V600E} mutant MaMel cells.

(**A**) Representative flow cytometric analysis of EdU and PI intracellular co-staining, defining the cell cycle phases G0/G1, S, and G2/M. (**B**) Quantification of frequencies of the cells in cell cycle phases shown in A. Data from five independent experiments are shown (n = 5). (**C**) Representative analysis of MaMel cell viability under inhibition with vemurafenib (PLX; concentration range: 200 µM - 0.8 nM). (**D**) IC50 values obtained from MTT assays on MaMel cells under inhibition with vemurafenib. Data from five independent experiments are shown (n = 5). (**E**) Representative analysis of MaMel cell viability under inhibition with trametinib (TR; concentration range: 500 nM - 0.002 nM). (**F**) Quantification of MaMel cell viability under 500 nM of trametinib. Data from three independent experiments are shown (n = 3). (**G**) Representative analysis of MaMel cell viability under inhibition with dactolisib (BEZ; concentration range: 10 µg/ml - 0.07 ng/ml). (**H**) IC50 values obtained from MTT assays on MaMel cells under inhibition with dactolisib. Data from three independent experiments are shown (n = 3). Data shown in C-H were acquired using MTT assays. All bar plots show the mean ± SD of the individual experiments. *p < 0.05, **p < 0.01, ***p < 0.001, ****p < 0.0001.

Fig. S2

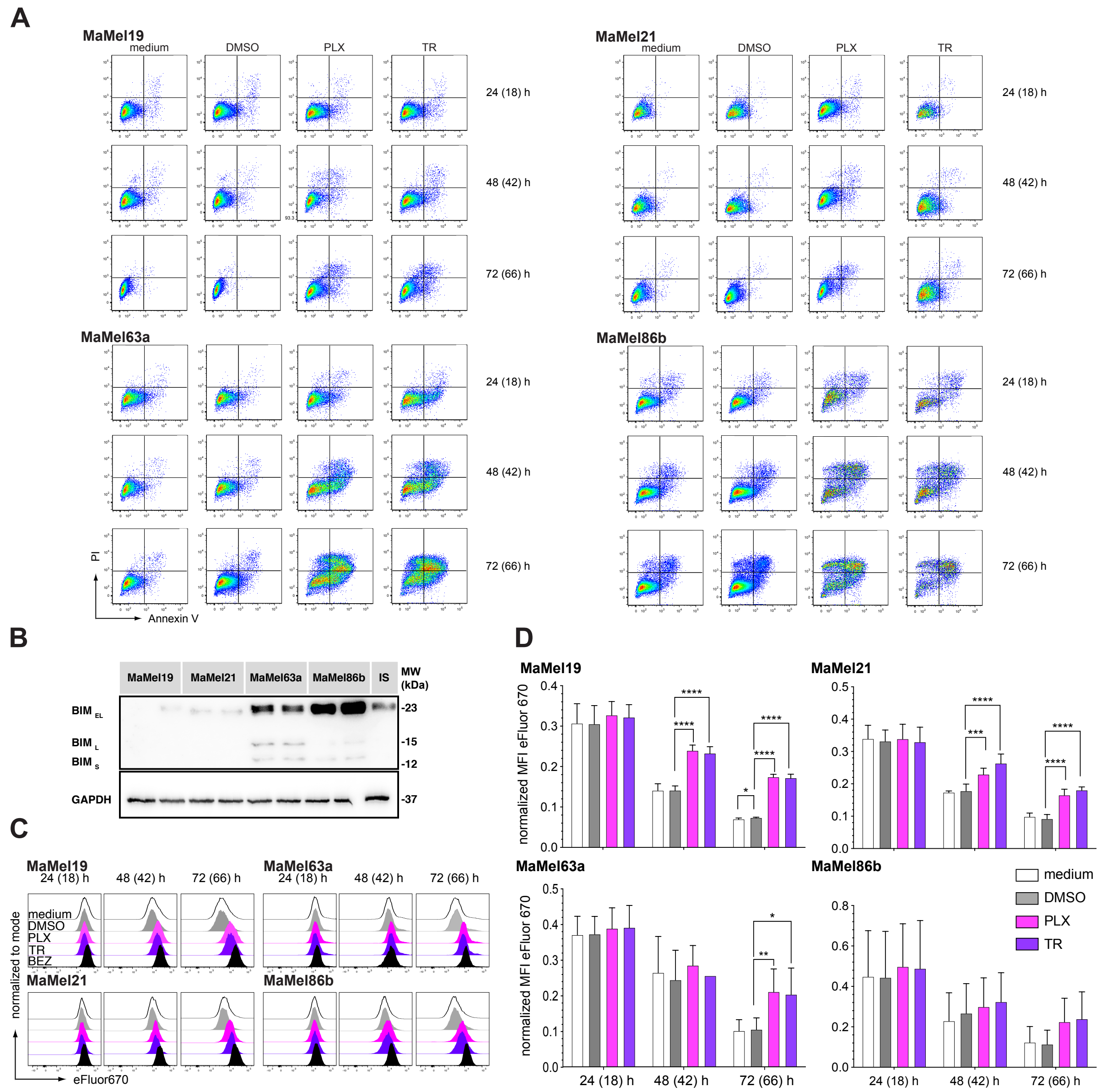

Fig. S2. Apoptotic and proliferative response of BRAF^{V600E} mutant MaMel cells under inhibition.

(**A**) Representative flow cytometric analysis of Annexin V and PI co-staining, defining the viable (Annexin V – PI negative; lower left quadrant) and apoptotic/necrotic cells in MaMel cell lines under the indicated treatment conditions for three different time points. (**B**) Western blot analysis of BIM expression in MaMel cell lines. Three BIM isoforms (EL, L, and S) are resolved. GAPDH served as loading control. (**C**) Representative flow cytometric analysis of cell proliferation rate using the eFluor 670 dye in MaMel cells under the indicated treatment conditions for three different time points. Cell proliferation is detected as a reduction of the fluorescence intensity of eFluor 670 dye. (**D**) Quantification of the cell proliferation rate of MaMel cells under inhibition, detected as a reduction of the median fluorescence intensity (MFI) of the eFluor 670 dye. Data from three independent experiments are shown (n = 3). Time points outside the brackets indicate the total time of cells in culture; time points in brackets indicate the inhibition time. All bar plots show the mean ± SD of the individual experiments. BEZ = dactolisib, PLX = vemurafenib, TR = trametinib. *$p < 0.05$, **$p < 0.01$, ***$p < 0.001$, ****$p < 0.0001$.

Fig. S3

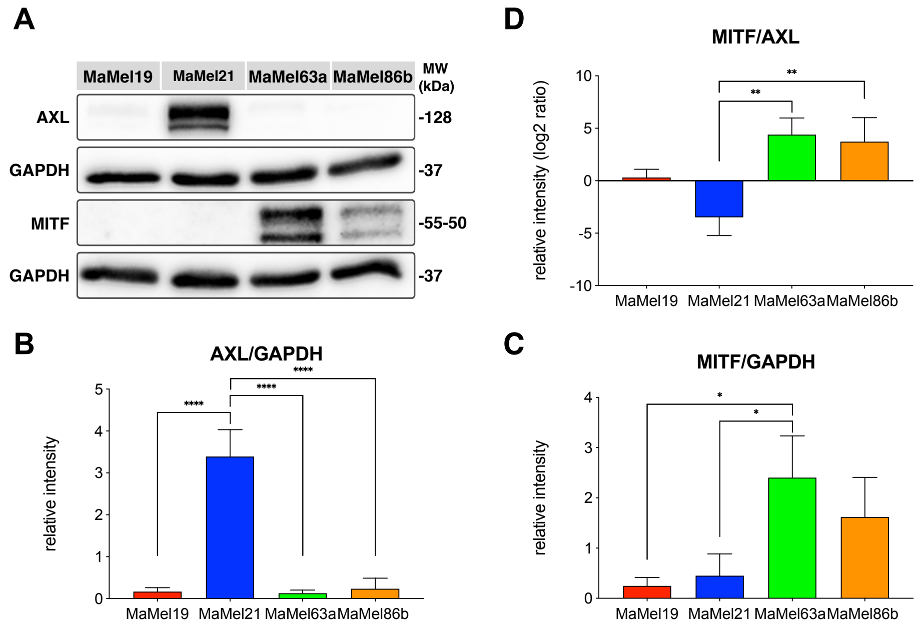

Fig. S3. MITF/AXL ratios in BRAF^{V600E} mutant MaMel cells.

(**A**) Representative Western blot analysis of AXL and MITF expression in MaMel cell lines. GAPDH served as a loading control. (**B**) Densitometric quantification of AXL and, (**C**) MITF. Optical density was normalized to the corresponding loading control. (**D**) Ratio of MITF/AXL intensities. All bar plots show the mean ± SD of three independent experiments (n = 3). *$p < 0.05$, **$p < 0.01$, ***$p < 0.001$, ****$p < 0.0001$.

Fig. S4

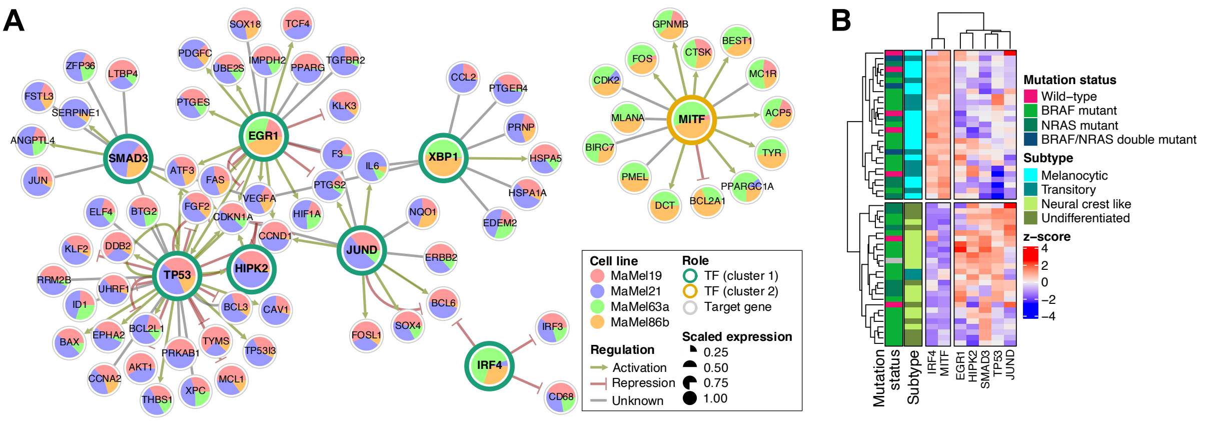

Fig. S4. Over-represented targets of transcription factors within groups of genes correlated with the degree of resistance in BRAF^{V600E} mutant MaMel cells.

(**A**) Network of transcription factors (TF) and their target genes, enriched within clusters of genes increasing (cluster 1) or decreasing (cluster 2) their expression along with higher levels of resistance to MAPK/ERK pathway inhibition. The nodes are colored according to the scaled expression of the respective gene for the different MaMel cell lines. The arrow type and color of the edges indicate the predicted regulation exerted by the TF over the corresponding target gene, according to TRRUST v2 database [13]. (**B**) Normalized expression (z-score) from TF in 53 melanoma cell lines from Tsoi et al. [30], showing different NRAS/BRAF mutation status and degrees of differentiation.

Fig. S5

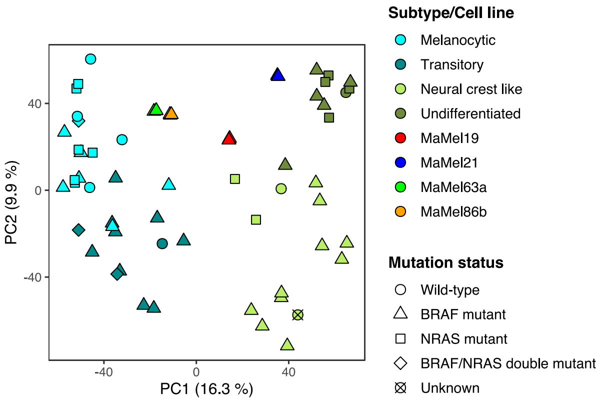

Fig. S5. Transcriptome profiles of melanoma cells with different NRAS/BRAF mutation status and degrees of differentiation.

Transcriptomes of MaMel cells are projected into the PCA space of the cohort of 53 melanoma cell lines from Tsoi et al. [30].

Fig. S6

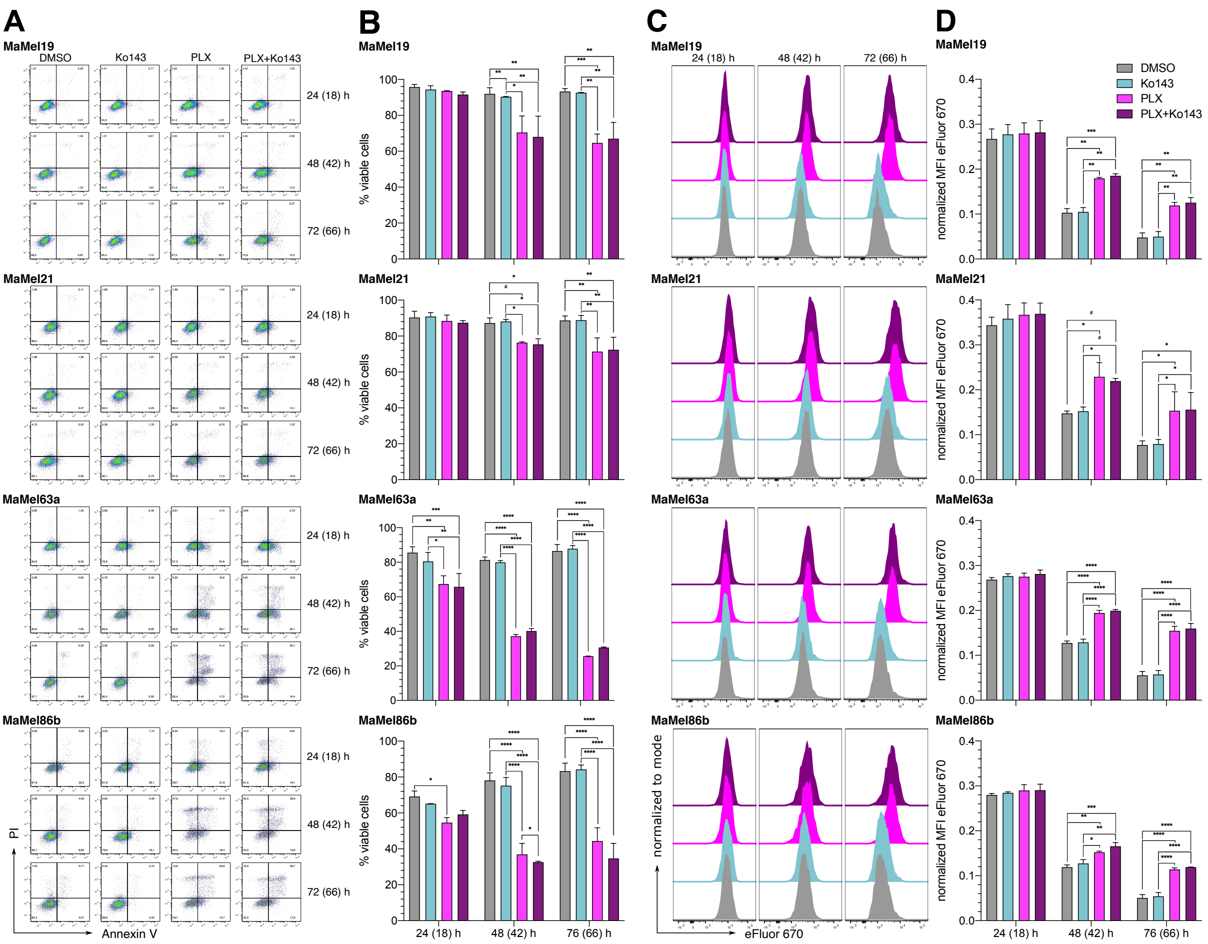

Fig. S6. Apoptotic and proliferative response of MaMel cells under combined BRAF and ABC transporter inhibition.

(**A**) Representative flow cytometric analysis of Annexin V and PI co-staining, defining the viable (Annexin V – PI negative; lower left quadrant) and apoptotic/necrotic cells in MaMel cell lines under the indicated treatment conditions for three different time points. (**B**) Quantification of the percentage of viable cells under inhibition, detected by Annexin V and PI co-staining. (**C**) Representative flow cytometric analysis of cell proliferation rate using the eFluor 670 dye in MaMel cells under the indicated treatment conditions for three different time points. Cell proliferation is detected as a reduction of the fluorescence intensity of eFluor 670 dye. (**D**) Quantification of the cell proliferation rate of MaMel cells under inhibition, detected as a reduction of the median fluorescence intensity (MFI) of the eFluor 670 dye. Time points outside the brackets indicate the total time of cells in culture; time points in brackets indicate the inhibition time. All bar plots show the mean ± SD of two independent experiments (n = 2). PLX = vemurafenib, Ko143 = ABCG2 inhibitor. $^{\#}p < 0.1$, $^{*}p < 0.05$, $^{**}p < 0.01$, $^{***}p < 0.001$, $^{****}p < 0.0001$.

Fig. S7

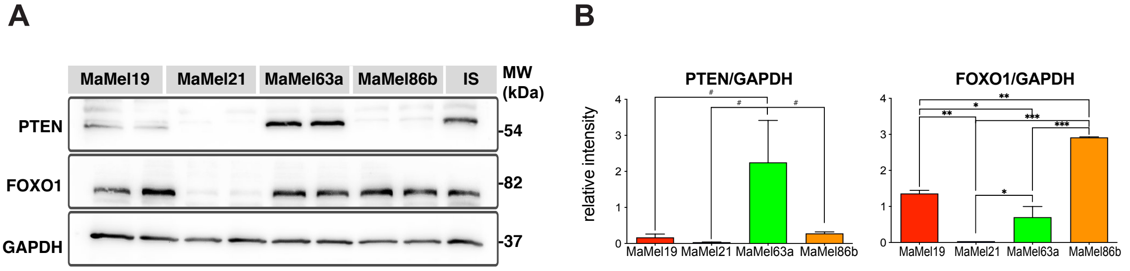

Fig. S7. PTEN and FOXO1 expression in BRAF^{V600E} mutant MaMel cells.

(**A**) Representative Western blot analysis of PTEN and FOXO1 expression in MaMel cell lines. GAPDH served as a loading control. (**B**) Densitometric quantification of two independent experiments (n = 2). Optical density was normalized to the corresponding loading control and internal standard (IS). Bar plots show the mean ± SD of the experiments. $^{\#}p < 0.1$, $^{*}p < 0.05$, $^{**}p < 0.01$, $^{***}p < 0.001$.
